# Process of distant lymph node metastasis in colorectal carcinoma: Implication of extracapsular invasion of lymph node metastasis

**DOI:** 10.1186/1471-2407-11-216

**Published:** 2011-06-02

**Authors:** Takaaki Fujii, Yuichi Tabe, Reina Yajima, Satoru Yamaguchi, Soichi Tsutsumi, Takayuki Asao, Hiroyuki Kuwano

**Affiliations:** 1 Department of General Surgical Science, Graduate School of Medicine, Gunma University, (3-39-22 Showa-machi, Maebashi), Gunma, (371-8511), Japan

## Abstract

**Background:**

We previously demonstrated that extracapsular invasion (ECI) at a metastatic sentinel node was significantly associated with the presence of positive non-sentinel nodes in patients with breast cancer. However, the mechanism of metastatic spreading of tumor cells to distant lymph nodes in patients with colorectal carcinoma is not fully understood. In this study, we investigated the factors that may determine the likelihood of additional regional lymph node metastasis when metastasis is found in nodes at the N1 site in colorectal cancer, especially focusing on the presence of ECI.

**Methods:**

Two hundred and twenty-eight consecutive patients who underwent colorectal resection were identified for inclusion in this study, of which 37 (16.2%) had positive lymph nodes at the N1 site. Six of these 37 cases had additional metastasis in N2 site lymph nodes. We reviewed the clinicopathological features of these cases and performed statistical analysis of the data.

**Results:**

In the univariate analysis ECI at the N1 site was the only factor significantly associated with the presence of cancer cells in the N2 site. Other factors, including number of positive lymph nodes, lymphovascular invasion of the primary tumor, tumor size and tumor depth of invasion, were not associated with metastatic involvement at the N2 site.

**Conclusions:**

Our results suggest that the presence of ECI at metastatic lymph nodes at the N1 site is correlated with further metastasis at the N2 site. These findings imply the possibility that ECI might indicate the ability of colorectal tumor cells to disseminate to distant lymph nodes.

## Background

Lymph node status is one of the most important prognostic factors for colorectal carcinoma. Tumor cells invade the lymphatic vessels, which enables tumor cells to penetrate into the lymphatic system. Both experimental tumor models and human clinicopathologic data indicate that growth of lymphatic vessels near solid tumors is often associated with lymph node metastasis [1,2]. However, little is known about the mechanism or process of metastatic spreading of tumor cells to distant regional lymph nodes in patients with colorectal carcinoma with positive lymph nodes.

We previously reported that the presence of an extracapsular invasion (ECI) at sentinel lymph nodes was a strong predictor of residual axillary disease, or non-sentinel lymph node metastasis in breast cancer [3,4], and the identification of the ECI of the metastatic lymph nodes has been reported as a prognostic factor in patients with colorectal cancer [5-7]. Thus, the purpose of this study was to investigate the correlation between the presence of ECI of positive lymph nodes at the N1 site and nodal metastasis at the N2 site in cases with resectable colorectal cancer.

## Methods

Two hundred and twenty-eight consecutive patients who underwent colorectal resection in the Department of General Surgical Science, Graduate School of Medicine, Gunma University, from January 2007 to December 2009 were identified for inclusion in this study. Patients with recurrent cases (2 cases), neo-adjuvant chemotherapy and radiation (33 cases), skipping lymph node metastases (9 cases) or incomplete clinical information were excluded. Of these eligible cases, 37 (16.2%) had positive lymph nodes at n1 (nodal metastases at the N1 site) and were analyzed in this study. The regional lymph nodes were classified according to criteria of the Japanese Research Society for Cancer of the Colon and Rectum: pericolic lymph nodes that lie along the marginal arteries (N1 site); and central intermediate lymph nodes that lie along the ileocolic, right colic, middle colic, left colic, sigmoid, and inferior mesenteric arteries from the origin of the last sigmoid artery to the origin of the left colic artery (N2 site) [8,9]. Informed consent for study participation was obtained from all patients.

**Figure 1:**
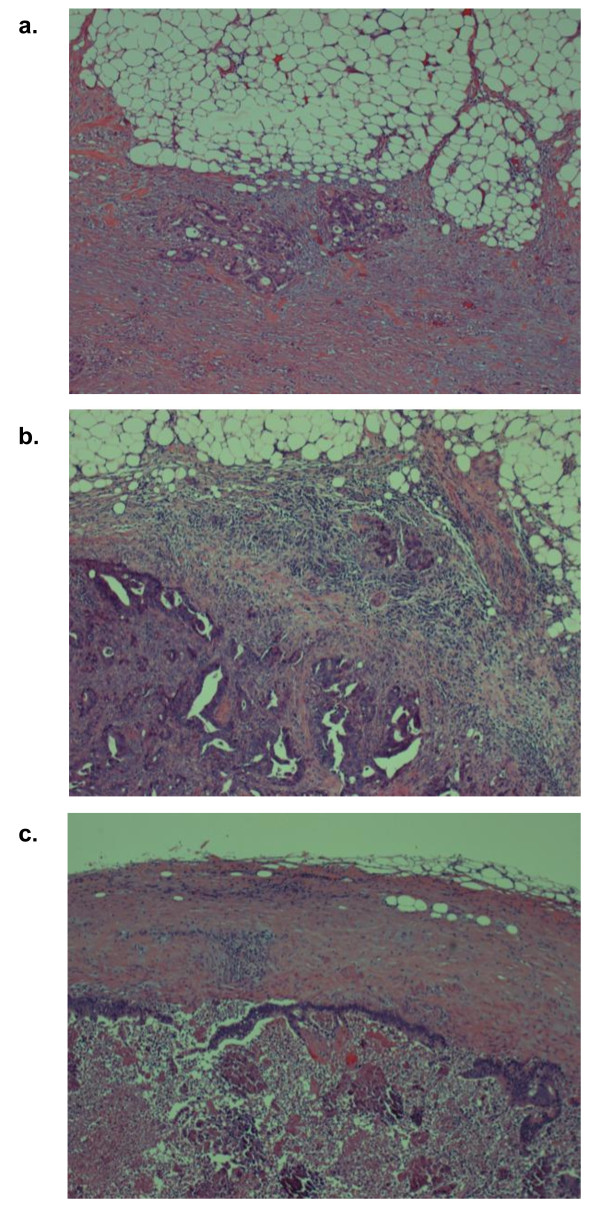
**Histological examples of absence or presence of extracapsular invasion (ECI) at metastatic lymph node**. **(a,b) **Case with present of ECI at metastatic lymph nodes; the tumor cells invade through the capsule of the lymph node. **(c) **Case with absent of ECI at metastatic lymph node; lymph node was contained metastatic foci, however, tumor cells did not invade the capsule of the lymph node.

Primary tumor size, tumor depth of invasion, age, sex, histological type, lymphovascular invasion at the primary tumor site, number of metastatic lymph nodes, extracapsular invasion (ECI) at positive lymph nodes and serum tumor marker (carcinoembryonic antigen, CEA) were tested as possible predictors of lymph node metastases at the N2 site. ECI was defined as extra capsular growth of tumor cells, invasion of perinodal fat or extranodal location of tumor cells (Figure 1). All resected lymph node were bisected in largest dimension. All sections were paraffin-embedded and stained with hematoxylin and eosin. Fisher's exact test and Student's *t*-test were used to compare metastatic and unaffected N2 site lymph nodes groups. Differences were considered to be significant when *P *< 0.05. The multivariate analysis was conducted using a regression model. To test the independence of the factors, the variables in the univariate analyses were entered into a multiple regression analysis with a likelihood of P < 0.05.

## Results

The 37 cases with metastatic lymph nodes at the N1 site were divided into two groups based on the presence or absence of metastasis at N2 site lymph nodes. Among 37 cases with positive lymph nodes at the N1 site, 6 (16.2%) had metastatic lymph nodes at the N2 site. Table 1 shows the patients and tumor characteristics. The mean age was 65.6 ± 14.7 years and the mean primary tumor size was 41.6 ± 18.2 mm. Table 1 also summarizes the results of the univariate analysis conducted to determine the relationship between the clinicopathologic variables and the presence of N2 site metastasis. Age, histological type, number of positive lymph nodes, lymphovascular invasion of the primary tumor, tumor size, tumor depth of invasion and serum CEA were not predictors of metastatic involvement at the N2 site. In the univariate analysis ECI at the N1 site was the only factor significantly associated with the presence of cancer cells in the N2 site (*P *= 0.005). Multivariate subgroup analysis of the association between these factors and recurrent disease showed that only the presence of ECI was independently associated with recurrent disease (RR = 2.30, 95%; CI 1.25-4.22; *P *= 0.015). In the group negative for metastasis at the N2 site, there were 9 patients with ECI. The number of positive lymph nodes was 3.89 ± 2.88 in those patients. One of the 9 cases with ECI without N2 metastasis had mucinous adenocarinoma and one of the 9 cases had diffuse matastasis at the N2 site. On the other hand, 1 of 6 cases with ECI and N2 metastasis had diffuse metastasis at the N2 site. There were no significant difference between the cases with ECI and N2 metastasis and the cases with ECI and without N2 metastasis on clinicopathological findings. However, among 9 cases with ECI and without N2 metastasis, 1 (11.1%) had disease recurrence and among 6 cases with ECI and N2 metastasis, 4 (66.7%) had recurrence.

**Table 1:** Patients characteristics and clinicopathologic features associated with further lymph node metastases at the N2 site

The N2 site metastasis	Positive	Negative	
Patients (n)	6	31	P value
Age (y ± SD)	71.5 ± 14.9	64.5 ± 14.4	0.301
Gender (M/F, n)	2/4	22/9	0.193
Location (Colon/Rectum, n)	5/1	21/10	0.782
Histrogical type (tub1-2/muc, n)	5/1	30/1	0.729
Tumor size (mm)	49.5 ± 14.1	40.0 ± 18.5	0.256
pT category (T1,2/T3,4, n)	0/6	7/24	0.470
Number of positive LNs (n)	3.5 ± 1.0	2.2 ± 2.0	0.149
ECI (n)	6	9	0.005
Lymphovascular invasion (n)	6	30	0.353
CEA (3.0<, n)	2	10	0.671

LN, lymph node; ECI, extracapsular invasion.

## Discussion

Lymph node metastasis is an important prognostic factor in patients with colorectal cancer, and many studies have indicated that the location and number of metastatic nodes affect prognosis [5,10-12]. Many studies have described the risk factors of lymph node metastases [1,2,13], but information regarding the mechanism or process of the metastatic spread of tumor cells to distant regional lymph nodes in patients with colorectal carcinoma is sparse at present. The key observations made in this study can be summarized as follows: In our cases with resected colorectal cancer with metastatic lymph nodes, the presence of ECI at positive lymph nodes at the N1 site was significantly associated with lymph node metastasis at the N2 site. This finding suggests that ECI may be a key process following distant lymph node metastasis.

Previous studies have demonstrated and confirmed that the presence of ECI at metastatic lymph nodes is significantly related to prognosis in not only colorectal carcinoma but also in various other types of carcinoma [4-7,14-17]. The ability of metastatic nodes to recruit degradation factors that permit cancer cells to break through the lymph node capsule is indicative of a very aggressive cancer. These studies imply that ECI is a biologic marker of aggressive nodal disease. We previously demonstrated that ECI at metastatic sentinel nodes in breast cancer was strongly associated with non-sentinel nodes metastasis [3]. These findings essentially support our findings; the presence of ECI of positive lymph nodes is significantly related to the nodal spread of the tumor cells in colorectal cancer patients. Tumor cells invade the lymphatic vessels, which enables the tumor cells to penetrate into the lymphatic system. Furthermore, in the group negative for metastasis at the N2 site there were 9 patients with ECI (Table 1), and the presence of ECI may be a clear sign of more aggressive disease, as those 9 patients had a greater total number of positive lymph nodes than did the patients without ECI (*P *< 0.05).

It is clear that the successful clinical application of sentinel lymph node biopsy used in cases of breast cancer and melanoma cannot simply be transferred into colorectal cancer treatment [18,19]. Sentinel lymph node biopsy in cases with colorectal cancer is a controversial issue, and we have sometimes experienced cases of colorectal cancer with skipping lymph node metastasis, in which distant nodes were positive but those closer to the tumor were negative. Skipping nodal metastases in colorectal carcinoma suggested the possibility of a bypass flow that has not been generally recognized [20]. In our series, skipping nodal metastasis was observed in 9 cases among the 46 cases with positive lymph nodes. Therefore, the presence of ECI at a positive lymph node might explain in part the mechanism or process to disseminate to distant regional lymph nodes, and there thought to be other mechanism, including skipping nodal metastasis.

This study has potential limitations. The major limitation of our study is that we used retrospective methods of data collection. In addition, the number of cases in our study was relatively small. However, the clinical implications of this data are very important, and these findings serve to emphasize that ECI at metastatic lymph nodes is one of the processes or mechanisms of distant lymph node dissemination and is a biologic marker of aggressive nodal disease. Additional research is needed to explore other patterns or mechanisms of lymph node spreading.

## Conclusions

In conclusion, we have demonstrated that ECI at metastatic lymph nodes at the N1 site is related distant regional lymph node metastasis at the N2 site in colorectal cancer, which might represent the ability or process of colorectal tumor cells to disseminate to distant lymph nodes. Analyses of data from large randomized trials or experimental data are warranted to evaluate this relationship between ECI and spreading of lymph node metastasis.

## Competing interests

The authors declare that they have no competing interests.

## Authors' contributions

TF designed the study, performed the majority of experiments and wrote the manuscript; YT, RY, SY, ST, TA provided the collection of all the human material; HK involved in editing the manuscript. This manuscript was read and approved the submission by all coauthors.

## Pre-publication history

The pre-publication history for this paper can be accessed here:

http://www.biomedcentral.com/1471-2407/11/216/prepub
